# Hyperglycemia-induced VEGF and ROS production in retinal cells is inhibited by the mTOR inhibitor, rapamycin

**DOI:** 10.1038/s41598-021-81482-3

**Published:** 2021-01-21

**Authors:** Teruyo Kida, Hidehiro Oku, Sho Osuka, Taeko Horie, Tsunehiko Ikeda

**Affiliations:** Department of Ophthalmology, Osaka Medical College, 2-7 Daigaku-machi, Takatsuki-City, Osaka 569-8686 Japan

**Keywords:** Eye diseases, Medical research

## Abstract

Determine the impact of the mTOR inhibitor, rapamycin, on the hyperglycemia-induced expression of vascular endothelial growth factor (VEGF) and the production of reactive oxygen species (ROS) in retinal cells. Rats made hyperglycemic for 8 weeks by streptozotocin, as well as control rats, received i.p. rapamycin (1 mg/kg) for 3 days prior to immunostaining of their retinas with anti-VEGF and anti-glial fibrillary acidic protein (GFAP) and measuring retinal protein levels of VEGF and GFAP by Western blotting. In other experiments, flow cytometry analysis of ethidium fluorescence determined intracellular ROS levels in the absence or presence of rapamycin (1 μM) under normoglycemic (5.5 mM) and hyperglycemic (25 mM) conditions in a rat retinal Müller cell line (TR-MUL5) and primary human retinal microvascular endothelial cells (HRMECs). In the diabetic retina, VEGF was elevated and colocalized with the glial marker, GFAP, whose level was also elevated. Treatment with rapamycin inhibited the diabetes-induced VEGF and GFAP increases. We also found that raising extracellular glucose from 5.5 mM to 25 mM resulted in significant rapamycin-sensitive increases in the ROS levels of TR-MUL5 cells and HRMECs. In rat retina, rapamycin attenuates the diabetes-induced VEGF overexpression, and in cultured Müller cells and HRMECs, inhibits the hyperglycemia-induced boost ROS.

## Introduction

Hyperglycemia is the primary cause of vascular complications occurring in individuals with diabetes^1–3^. An important microvascular complication is sight-threatening diabetic retinopathy^4–6^ in which chronic hyperglycemia is associated with increased expression of vascular endothelial growth factor (VEGF), which is known to be synthesized in the retinal Müller glial cells, and elevated levels of reactive oxygen species (ROS), which are produced by retinal cells including the glia and vascular endothelium^7–12^.

These responses of the diabetic retina are likely to be interrelated since exposure to VEGF is known to boost the production of ROS by the Müller cells and also the cells of the vascular endothelium^13,14^. Even though the physiological roles of VEGF in the retina remain uncertain, its upregulation is well-established to also cause a breakdown of the blood-retinal barrier, whose integrity is essential for optimal retinal function. Furthermore, although redox signaling is plays a key role in the physiological regulation of blood flow^9,15,16^, ROS overproduction is thought to contribute to the endothelial dysfunction and neurodegeneration observed in diabetic retinopathy^3,17–19^.

In this study, we considered the working hypothesis that the mTOR signaling pathway plays a role in the pathological upregulation of retinal VEGF and ROS observed in the course of diabetes. Not only has elevated mTOR activity been observed in diabetes^20^, but the mTOR pathway is involved in the upregulation of VEGF. To begin assess our working hypothesis, we first performed immunohistochemistry to compare the expression of VEGF and the glial marker, GFAP, in the diabetic retina and the effects of rapamycin, an immunosuppressive and anti-cancer drug that is reported to suppress the action of mTOR^21,22^. We also used immunoblots to determine whether VEGF and GFAP expression were increased in the retina of a streptozotocin (STZ)-induced rat model of diabetes. In addition, we conducted a flow cytometry study to determine the rapamycin effects on ROS levels in cultured TR-MUL5 cells, a rat Müller cell line, and primary human retinal microvascular endothelial cells (HRMECs) as well, following exposure to physiological (5.5 mM) and high glucose (25 mM) conditions.

In this study we show that rapamycin effectively attenuates the hyperglycemia-induced overexpression of VEGF and GFAP in the rat retina. In addition, this mTOR inhibitor suppressed the high glucose-induced increases in ROS in cultured Müller cells as well as HRMECs whose dysfunction and degeneration is a well-established feature of diabetic retinopathy.

## Materials and methods

### Animals

Twenty-four 9-week-old male Wistar rats weighing between 180 and 200 g were purchased from Japan SLC Inc. (Shizuoka, Japan) and housed in an air-conditioned room at approximately 23 °C with 60% humidity. They were maintained on a 12-h light/dark cycle and were provided with food and water ad libitum. They were handled in accordance with the ARVO Statement for the Use of Animals in Ophthalmic and Vision Research. Our experimental protocols conformed to the Animal Research: Reporting In Vivo Experiments (ARRIVE) guidelines^23^ and was approved by the Osaka Medical College Committee on the Use and Care of Animals (Approval number: 2019-100).

### Chemicals

All chemicals were obtained from Sigma-Aldrich (St. Louis, MO, USA) unless otherwise specified. Rapamycin was purchased from Tokyo Chemical Industry (Tokyo, Japan). A rapamycin dose of 1 mg/kg has been confirmed as neuroprotective and renoprotective^24,25^, so we chose this concentration for our experiments. In addition, a rapamycin concentration of 1 μM, which was converted from the dose of the rapamycin confirmed in vivo study, was used for in vitro study.

### Anesthesia and euthanasia

All procedures were performed anesthesia using intraperitoneal injection of medetomidine (0.75 mg/kg), midazolam (4 mg/kg), and butorphanol (5 mg/kg) as described previously^26^. Rats were euthanized by exposure to CO_2_ with wood shaving bedding.

### Induction of diabetes

Diabetic and non-diabetic rats were produced as described previously^27^. Diabetes was induced by administering a single 60 mg/kg intraperitoneal injection of streptozotocin in 10 mM sodium citrate buffer, pH 4.5, to each rat after an overnight fast. As controls, nondiabetic animals received an injection of citrate buffer only. Animals with blood glucose levels higher than 250 mg/dL at 24 h after injections were considered diabetic. All experiments were conducted 8 weeks after the induction of diabetes. The blood glucose was checked again at the time of the experiments to ensure that the rats showed hyperglycemia. The blood glucose level at the time of sacrifice was 384.8 ± 23.9 mg/dL.

### Intraperitoneal injection of rapamycin

Rapamycin (1 mg/kg) (Tokyo Chemical Industry, Tokyo, Japan) was injected intraperitoneally once a day for 3 days in STZ-induced diabetic and normoglycemic rats. Animals received general anesthesia as previously described, and perfused fixation was performed 24 h after the intraperitoneal injections.

### Immunohistochemistry of retinal slices

All procedures were performed as described previously^27^. Twelve rats were deeply anesthetized, and saline was perfused through the heart, followed by 4% paraformaldehyde (PFA) in 0.1 M phosphate-buffered saline (PBS, pH 7.4). The retinas were carefully removed and post-fixed in 4% PFA in PBS overnight. After washing with PBS, the retinal tissues were immersed in 30% sucrose for 2–3 days at 4 °C, embedded in OCT compound (Sakura Finetechnical, Tokyo, Japan), and cut into 10 μm-thick frozen sections with a cryostat. After blocking with 1% normal goat serum plus 1% BSA and 0.1% triton X-100 in PBS, the retinal sections were incubated overnight at 4 °C with the following primary antibodies: mouse monoclonal anti-VEGF (1:500, sc-7269, Santa Cruz Biotechnology, Inc., Dallas, TX, U.S.A.) and rabbit polyclonal anti-GFAP (1:500, AB5804, Merck Millipore, Billerica, MA, U.S.A.). These sections were incubated for 2 h at 37 °C with the appropriate Alexa 594- (A11012) or Alexa 488- (A11001) conjugated secondary antibodies (1∶500; Invitrogen, Carlsbad, CA, U.S.A.). The processed sections were photographed with a fluorescent microscope (BZ-X700, Keyence, Osaka, Japan) by using Z-scan of 35 images at 0.5 μm intervals in each retinal section under the same exposure time.

### Protein levels of VEGF and GFAP in retinas by western blotting

All procedures were performed as described previously^27,28^. Retinas were excised from the eyes and homogenized in lysis buffer containing 1 mM phenyl methanesulfonyl fluoride, 10 μM pepstatin A, 10 μM leupeptin, 10 μM aprotinin, 0.1% sodium dodecyl sulfate (SDS), 1% Nonidet P-40, 5% sodium deoxy cholate, 50 mM Tris–HCl (pH 7.6), and 150 mM sodium chloride. The suspension was centrifuged, and the total protein concentration in the supernatant was determined using the Lowry method (DC Protein Assay Reagent, Bio-Rad, Hercules, CA, USA). Samples were separated on a 10–12% SDS-polyacrylamide gel and blotted onto PVDF membranes. The membranes were then blocked with 5% skim milk in Tris-buffered saline, pH 7.4, with 0.1% Tween 20 (TBS-T) followed by an overnight incubation at 4 °C with a rabbit polyclonal anti-VEGF (1:1000, sc-507, Santa Cruz Biotechnology) or GFAP (1:1000, 04-1031, Merck Millipore). Tubulin (α-tubulin, 1:1000; Merck Millipore) was used as an internal control. The membranes were washed in TBS-T followed by incubation with a peroxidase-conjugated goat anti-rabbit (W401B) or mouse IgG (W402B, 1:2500, Promega, Madison, WI, USA) secondary antibody for 2 h at 37 °C. The protein bands were visualized following the addition of an ECL Plus Western blotting detection system (GE Healthcare Life Sciences, Little Chalfont, UK). Protein band densities were measured with a luminescent image analyzer (LAS-3000, Fujifilm, Tokyo, Japan). Relative protein levels were quantified using the embedded software (Multi Gauge version 3.0) and standardized according to α-tubulin protein levels.

### Cell cultures

A rat retinal Müller cell line (TR-MUL5) was obtained from Fact, Inc., Sendai, Japan^29,30^, and primary human retinal microvascular endothelial cells (HRMECs) were purchased from Cell Systems Corporation, Kirkland, WA, USA. The TR-MUL5 cells and the HRMECs were cultured in Dulbecco’s Modified Eagle’s Medium (DMEM) containing a physiological concentration of 5.5 mM glucose supplemented with 10% fetal bovine serum (FBS) at 37 °C in a humidified atmosphere of 5% CO_2_/air. TR-MUL5 cells at passages 18 and 21, and HRMECs at passages 4 and 6 were used in this study. These cells were maintained in DMEM that contained 5.5 mM glucose. Before reaching confluence, the media was changed to the control media (5.5 mM glucose in DMEM) or the high glucose media (25 mM glucose in DMEM, D6429) to compare ROS levels, as described later. Detailed procedures and treatment protocols for each experiment are described in subsequent sections.

### Immunostaining of cultured Müller cells

To determine the effect of the mTOR inhibitor rapamycin on the expression of VEGF and GFAP in the TR-MUL5 cells under high glucose condition, the cells that were incubated in high glucose (25 mM) media with and without rapamycin were examined by immunocytochemistry. After fixation by 4% formaldehyde, the cells were incubated with primary antibodies of mouse monoclonal anti-VEGF (sc-7269, 1:100, Santa Cruz Biotechnology, Inc., Dallas, TX, U.S.A.) and rabbit polyclonal anti-GFAP (AB5804, 1:100, Merck Millipore, Billerica, MA, U.S.A.) overnight at 4 °C. After rinsing by PBS and blocking, these cells were incubated for 2 h at room temperature in Alexa 594 (A11012) or Alexa 488- (A11001) conjugated to the appropriate secondary antibodies (Invitrogen, Carlsbad, CA, U.S.A.) diluted by 1:500. Nuclei were stained with 4′,6-diamidino-2-phenylindole (DAPI, 1:1000, Dojindo, Inc. Kumamoto, Japan). The processed samples were photographed with a fluorescence microscope (BZ-X700, Keyence, Osaka, Japan).

### Flow cytometry analyses of ROS formation in müller cells and HRMECs

We examined the generation of ROS in cultured Müller cells (TR-MUL5) and HRMECs by treating them with hydroethidine and measuring ethidium fluorescence by flow cytometry (EC800 cell analyzer, SONY, Tokyo, Japan). After a few passages of TR-MUL5 and HRMECs, the media was changed to the control media (5.5 mM glucose in DMEM) or the high glucose media (25 mM glucose in DMEM, D6429) with 10% fetal bovine serum (FBS). The cells were incubated overnight at 37 °C with or without rapamycin (1 μM) under physiological (5.5 mM) and high-glucose (25 mM) conditions for 48 h. The cells were cultured in a medium lacking 10% FBS and were serum-deprived overnight before the following FACS assay. Cells were harvested via trypsinization, centrifuged at 1000×*g* for 5 min, re-suspended in phenol red-free DMEM, and incubated with hydroethidine, a fluorogenic probe, for 20 min at 37 °C. Cell densities were adjusted to 2.0 × 10^5^ cells/mL. Hydroethidine is oxidized by ROS to form ethidium, a fluorescent product, which is then retained intracellularly to allow a semi-quantitative estimation of intracellular ROS levels^31,32^. The intracellular levels of ROS were measured using an EC800 cell analyzer at 488 nm excitation and 590–610 nm emission wavelengths. The acquisition and analysis software programs on the EC800 was used to acquire and quantify the fluorescence intensities. This assay was performed to determine the levels of ROS generated by the cultured cells in control and high glucose media in the presence or absence of rapamycin (1 μM). In addition, the cultured Müller cells (TR-MUL5) were also incubated overnight with or without VEGF (5 ng/mL) and rapamycin under physiological (5.5 mM) and high-glucose (25 mM) conditions to determine ROS production by VEGF and the effects of rapamycin on this ROS production. The ethidium fluorescence in TR-MUL5 cells exposed to VEGF and/or rapamycin under physiological (5.5 mM) and high-glucose (25 mM) conditions was also measured by flow cytometry.

### Statistical analyses

The means and standard deviation of the means were calculated. Unless otherwise noted, two-tailed Student’s *t*-tests were used. The level of significance was set at *P* < 0.05. Multiple groups in Figs. 4 and 5 were compared ANOVA analysis; Bonferroni correction.

### Ethical approval

All applicable guidelines of ARVO Statement for the care and use of animals were followed. Our experimental protocols conformed to the Animal Research: Reporting In Vivo Experiments (ARRIVE) guidelines^23^.

All procedures performed in studies involving animals were in accordance with the ethical standards of the Osaka Medical College Committee on the Use and Care of Animals (Approval number: 2019-100) at which the studies were conducted.

## Results

### Immunohistochemistry of retinal slices

**Figure 1:**
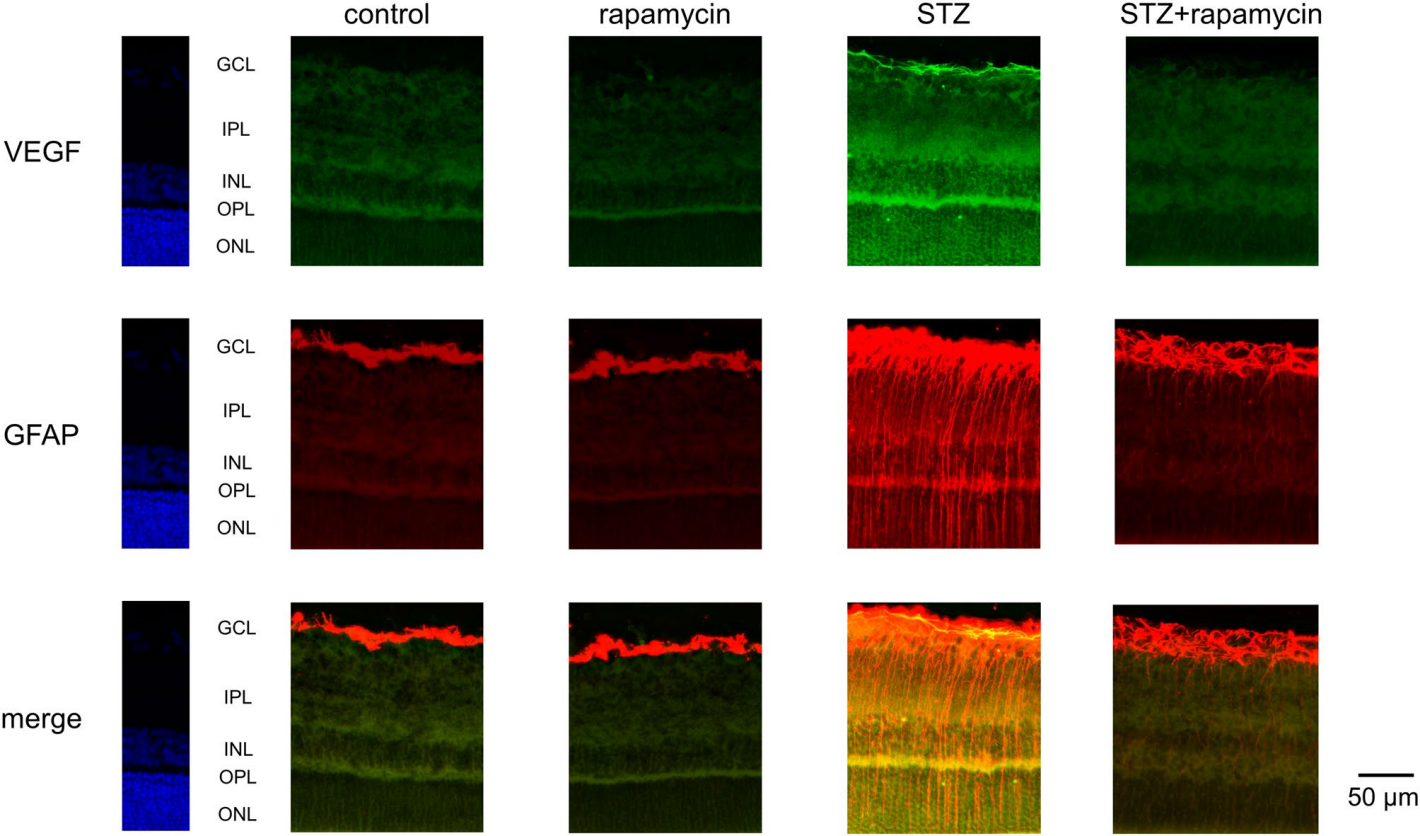
Representative photomicrographs of retinal tissues stained immunohistochemically with anti-VEGF (green) and anti-GFAP (red) antibodies. VEGF immunoreactivity was higher in the diabetic retina sections than in the control retinas, and expression of VEGF was colocalized with GFAP.

Compared with the nondiabetic control rats, immunoreactivity to VEGF was increased in the STZ-induced diabetic rats. As shown in Fig. 1, VEGF immunoreactivity was seemed to be higher in the diabetic rat retinas than in the controls. Injection of rapamycin into the nondiabetic rats seemed to decrease VEGF immunoreactivity. In the diabetic retina, VEGF expression seemed to be intensified mainly in these internal retinal layers. Rapamycin likely decreased the intensity in diabetic rats.

Immunoreactivity to GFAP seemed to be higher in the diabetic rat retina than in the control retina. The observed longitudinal pattern of immunoreactivity to GFAP in the inner plexiform layer (IPL), the inner nuclear layer (INL), the outer plexiform layer (OPL), and the outer nuclear layer (ONL) suggested that these GFAP expression may involve Müller cells. Double staining with VEGF and GFAP demonstrated that the expression of VEGF was co-localized with GFAP expression especially in the diabetic rat retinas. Rapamycin seemed to inhibit the increases in GFAP expression in the diabetic retina.

### Immunostaining of cultured Müller cells

**Figure 2:**
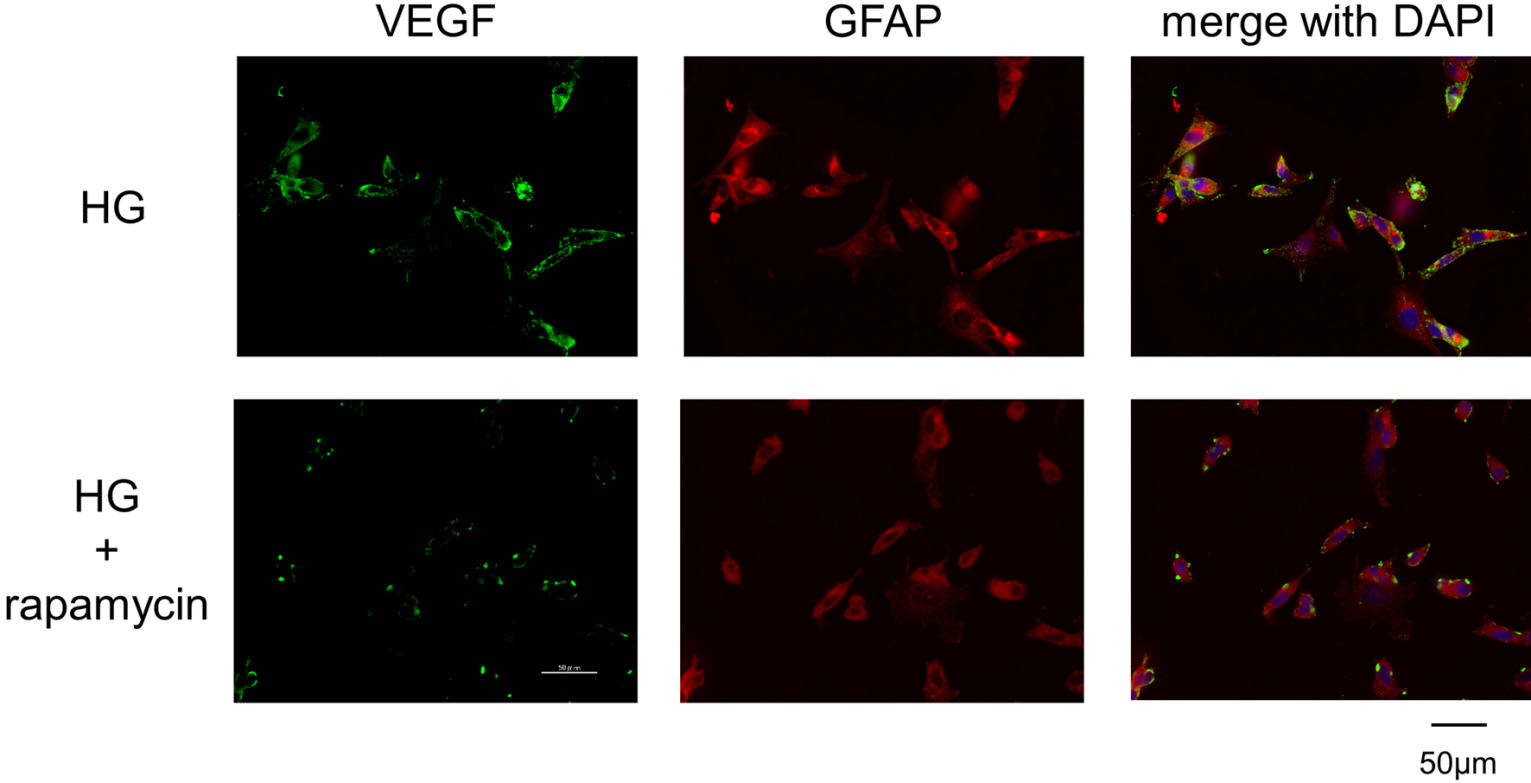
Immunostaining of VEGF and GFAP in TR-MUL5 cells. Expression of VEGF and GFAP in cells cultured in high glucose medium (HG) seemed to be reduced by the mTOR inhibitor rapamycin (HG + rapamycin).

Representative photomicrographs of immunostaining with anti-VEGF (green) and anti-GFAP (red) in the TR-MUL5 cells are shown in Fig. 2. Immunoreactivities to VEGF and GFAP in high glucose condition seemed to be depressed by the mTOR inhibitor rapamycin.

### Protein levels of VEGF and GFAP in retinas by western blotting

**Figure 3:**
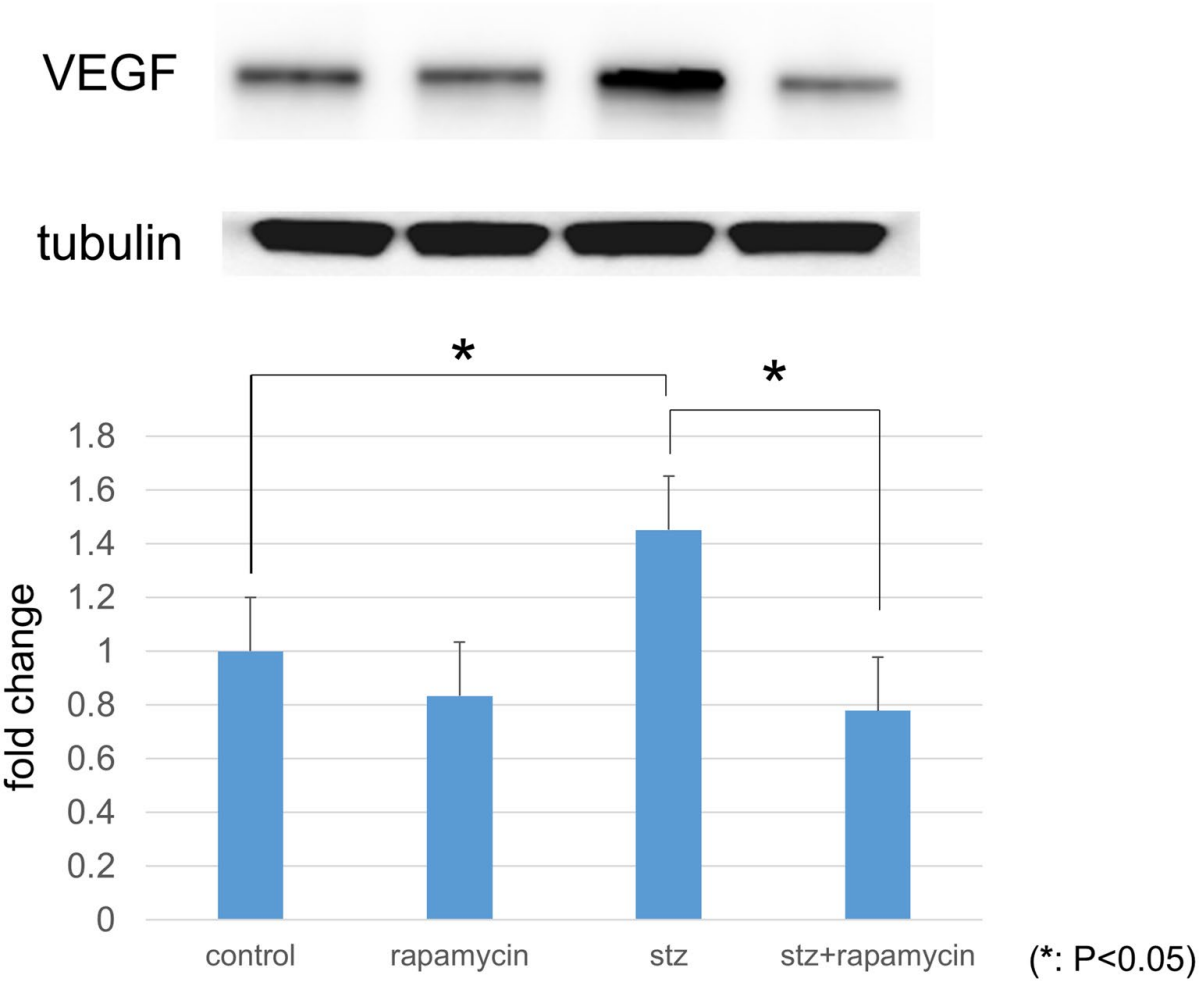
Protein levels of VEGF in retinas determined with Western blotting. VEGF expression was significantly higher in retinas obtained from STZ-induced diabetic rats than from nondiabetic controls. The increased VEGF expression was suppressed by treatment with the mTOR inhibitor rapamycin (*P* < 0.05, n = 3 each).

The protein levels of VEGF in the STZ-induced diabetic retina were significantly up-regulated to 145.2% compared to the control retina (*P* < 0.0001). These increases were significantly suppressed by rapamycin (*P* < 0.0001) (Fig. 3, n = 3 each).

**Figure 4:**
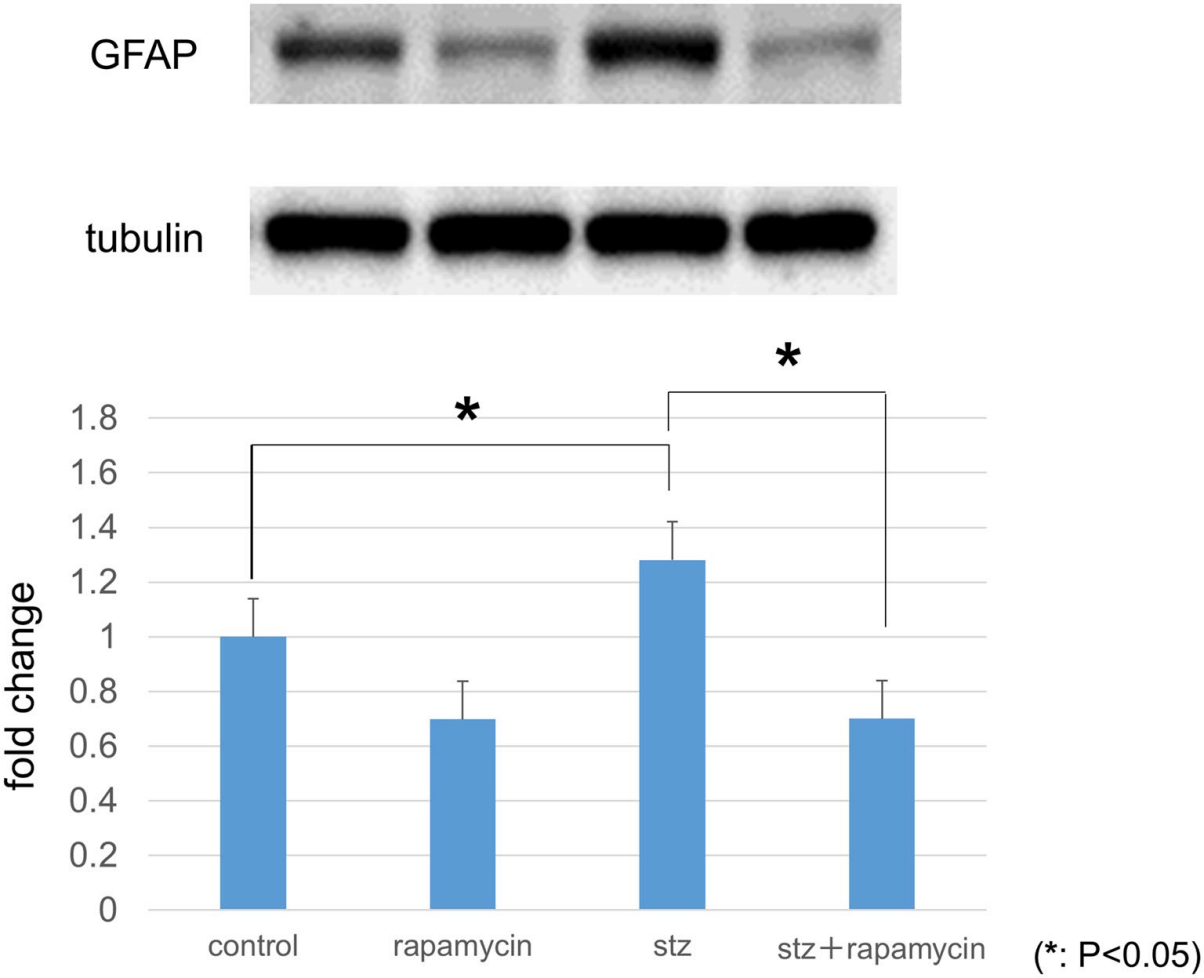
Protein levels of GFAP in retinas determined with Western blotting. GFAP expression was significantly higher in retinas obtained from STZ-induced diabetic rats than from nondiabetic controls. The increased GFAP expression was suppressed by rapamycin (*P* < 0.05, n = 3 each).

Protein levels of GFAP in the diabetic retina were significantly higher (28.1%) than in the control retina (*P* = 0.0004). The increases were significantly suppressed by rapamycin (*P* < 0.0001) (Fig. 4, n = 3 each).

### Flow cytometry analyses of intracellular production of ROS in TR-MUL5 and HRMECs

**Figure 5:**
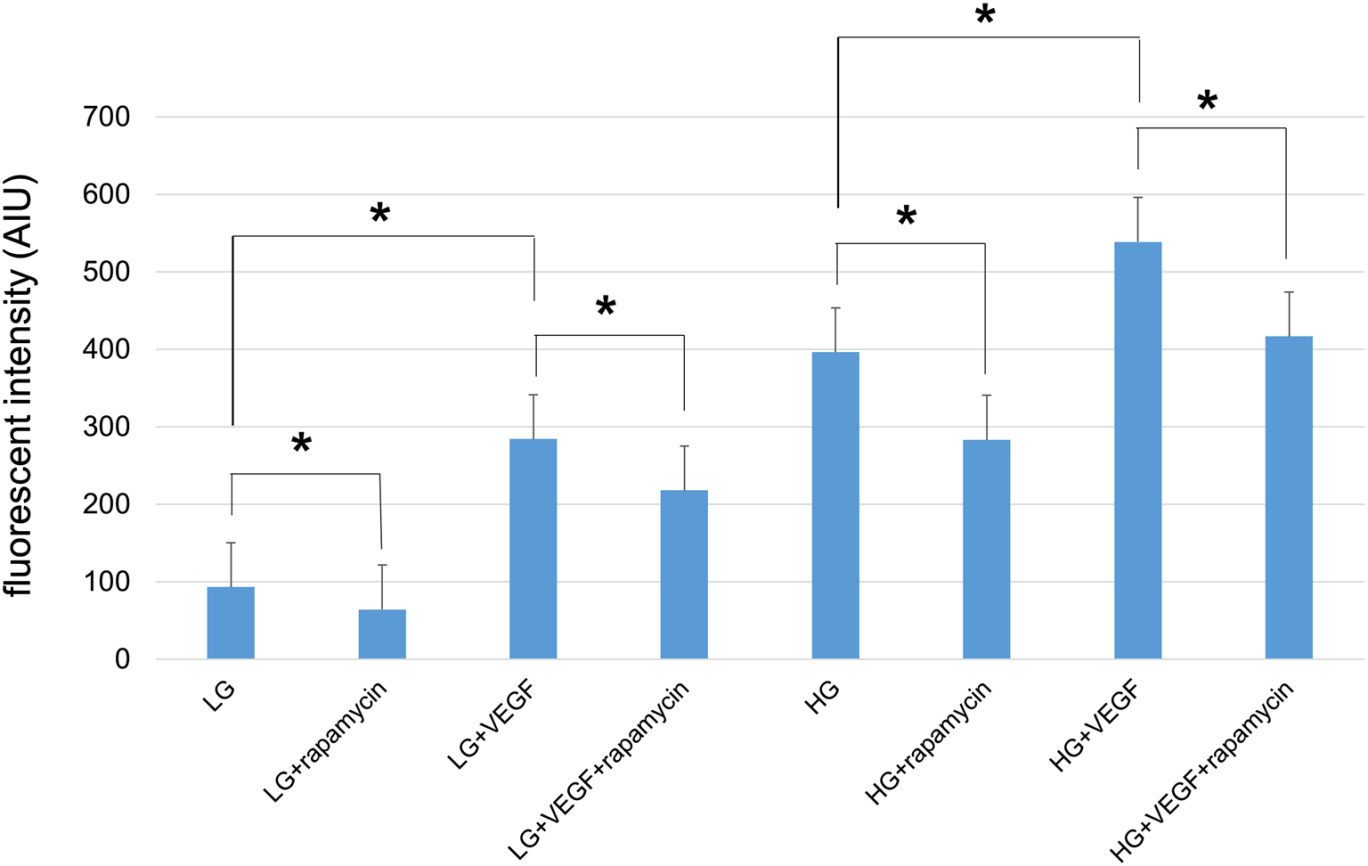
ROS levels in cultured Müller cells (TR-MUL5) exposed to rapamycin and/or VEGF under physiological (5.5 mM) and high-glucose (25 mM) conditions. Flow cytometry analyses showed significant increases in intracellular ROS levels in TR-MUL5 under a high-glucose condition, and these increases were significantly suppressed by rapamycin. In addition, flow cytometry analyses showed significant increases in intracellular levels of ROS in TR-MUL5 exposed to VEGF, and these increases were significantly suppressed by rapamycin (*P* < 0.0018, the Bonferroni correction).

Figure 5 shows the effects of rapamycin (1 μM) treatment on hydroethidine fluorescence in cultured rat Müller cells (TR-MUL5) under physiological (5.5 mM) and high-glucose (25 mM) conditions. Flow cytometry analyses showed significant increase in intracellular levels of ROS in TR-MUL5 under the high- glucose condition, and these increases were significantly suppressed by rapamycin. This figure also shows the effects of rapamycin on VEGF in ROS production of TR-MUL5 under physiological (5.5 mM) and high-glucose (25 mM) conditions. We performed this assay to determine the effect of rapamycin on the ROS production of cultured Müller cells (TR-MUL5) exposed to VEGF. Flow cytometry analyses showed significant increases in the intracellular levels of ROS in TR-MUL5 cells exposed to VEGF, and these increases were significantly suppressed by rapamycin (*P* < 0.0018, the Bonferroni correction).

**Figure 6:**
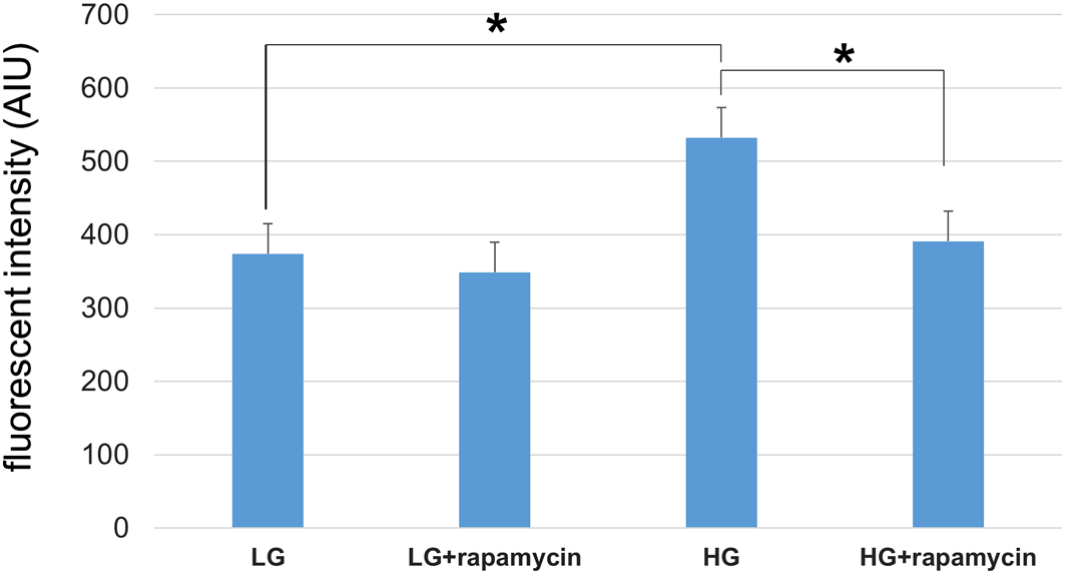
ROS levels in primary human retinal microvascular endothelial cells (HRMECs) exposed to rapamycin under physiological (5.5 mM) and high- glucose (25 mM) conditions. Flow cytometry analyses showed significant increases in intracellular ROS levels in HRMECs under a high-glucose condition, and these increases were significantly suppressed by rapamycin (*P* < 0.0083, the Bonferroni correction).

Figure 6 shows the intracellular ROS levels in HRMECs exposed to rapamycin under physiological (5.5 mM) and high-glucose (25 mM) conditions. Significant increases in the ROS levels in HRMECs under high-glucose condition were observed, and these increases were significantly suppressed by rapamycin (*P* < 0.0083, the Bonferroni correction).

## Discussion

A key finding of this study is that the diabetes-induced increase in VEGF expression in the diabetic rat retina is inhibited by the mTOR inhibitor, rapamycin. This enzymatic inhibitor also blocked the up-regulation of GFAP by glial cells of the diabetic retina. An additional novel observation is that rapamycin inhibits the hyperglycemia-induced increase in ROS in cultured Müller cells as well as HERMCs. We also found that under hyperglycemic conditions, the VEGF-induced increase in Müller cell ROS was also suppressed by rapamycin.

The increase in VEGF levels in the diabetic retina have been previously reported^19,27,33–35^, and this response was suppressed by rapamycin. Generally, mTOR coordinates protein synthesis, mitochondrial energy production^36^. In STZ-injected hyperglycemic rats which VEGF increases in the retina, this mTOR is amplified^20^. In the present study, rapamycin suppressed the increase of VEGF in the diabetic retina (Figs. 1, 2, 3) and prevented the hyperglycemia-induced ROS level in both cultured Müller cells (Fig. 5) and primary HRMECs (Fig. 6). This indicated that rapamycin seemed to decrease ROS levels by inhibiting the mTOR pathway in retinal Müller cells; however, we did not assess its effects on non-Müller rat retinal cells. Our results are consistent with co-localization being at least in part within the Müller cells, and it is not excluded from the present study that some non-Müller cells may also co-express VEGF and GFAP. In the retina, Müller cells and vascular endothelial cells might express mTOR.

ROS production, oxidative stress, and cellular death can be altered by several signaling pathways triggered by the hyperglycemia that causes diabetic retinopathy^37^. Our data indicated that VEGF-induced increase of the ROS levels in Müller cells under high-glucose conditions and this increase was suppressed by rapamycin. We also analyzed the intracellular ROS levels in primary HRMECs which have the potential to cause retinal vascularization observed in human diabetic retinopathy. Our findings indicate that rapamycin might be an effective alternative drug for the treatment of diabetic macular edema.

We also showed that GFAP, a glial marker, increased in the diabetic retina. Our findings showed that rapamycin can reduce the increase in GFAP expression in the diabetic retina, suggesting that mTOR inhibition may inhibit glial activation and/or proliferation. In non-diabetic rats, the effect of rapamycin on GFAP levels was observed as well. Rapamycin might inhibit the activity of retinal glia even also under the physiological condition. In diabetic retinopathy, neurodegeneration starts even at an early stage of the disorder where reactive gliosis plays a crucial role^17,38^. It is known that there are two types of mTOR, mTORC1 (mainly retinal ganglion cells) and mTORC2 (mainly glial cells) in the retina^39–41^. The effects of rapamycin in this study seem to be caused mainly by mTORC2 inhibition in the diabetic rat retina.

A limitation of this study is that although the chief focus was on oft-studied rat models of diabetes, it will be of importance for future analyses to extend analyses to the retinas of animals and humans exhibiting anatomical and functional manifestations of diabetic retinopathy. Another possible limitation is that although mTOR is reported to increase in the retinas of rats with experimental diabetes^20^, this remains to be confirmed in retinas of rats made hyperglycemic by STZ for 8 weeks. Thirdly, although FACS was used to quantify ROS in cultured retinal Müller cells and HRMECs, it remains to be demonstrated in future studies that rapamycin suppresses excessive ROS production in vivo within the diabetic retina.

In conclusion, our findings from in vivo and in vitro experiments showed that the mTOR inhibitor, rapamycin, blocks the up-regulation of VEGF and GFAP in the diabetic rat retina and also inhibits the hyperglycemia-induced increase in ROS in cultured Müller cells and HERMCs. These findings suggest that rapamycin may be a potential candidate drug for ameliorating retinal complications in diabetes.

## Supplementary information

Supplementary Information
