# Supplementary material for: Hyperglycemia-induced VEGF and ROS production in retinal cells is inhibited by the mTOR inhibitor, rapamycin
Source: Sci Rep. 2021 Jan 21;11:1885. doi: 10.1038/s41598-021-81482-3 (PMC7820225; doi:10.1038/s41598-021-81482-3)

**Hyperglycemia-induced VEGF and ROS production in retinal cells is inhibited by the mTOR inhibitor, rapamycin**

**Authors:**

Teruyo Kida ^1^*, Hidehiro Oku ^1^, Sho Osuka ^1^, Taeko Horie ^1^, and Tsunehiko Ikeda ^1^

^1^: Department of Ophthalmology, Osaka Medical College, Takatsuki-City, Osaka, Japan

***Corresponding Author:**

Teruyo Kida, MD, PhD

Department of Ophthalmology, Osaka Medical College,

2-7 Daigaku-machi, Takatsuki,

Osaka 569-8686 JAPAN

Tel. +81-72-683-1221

Fax. +81-72-681-8195

Email: [opt038@osaka-med.ac.jp](mailto:opt038@osaka-med.ac.jp)


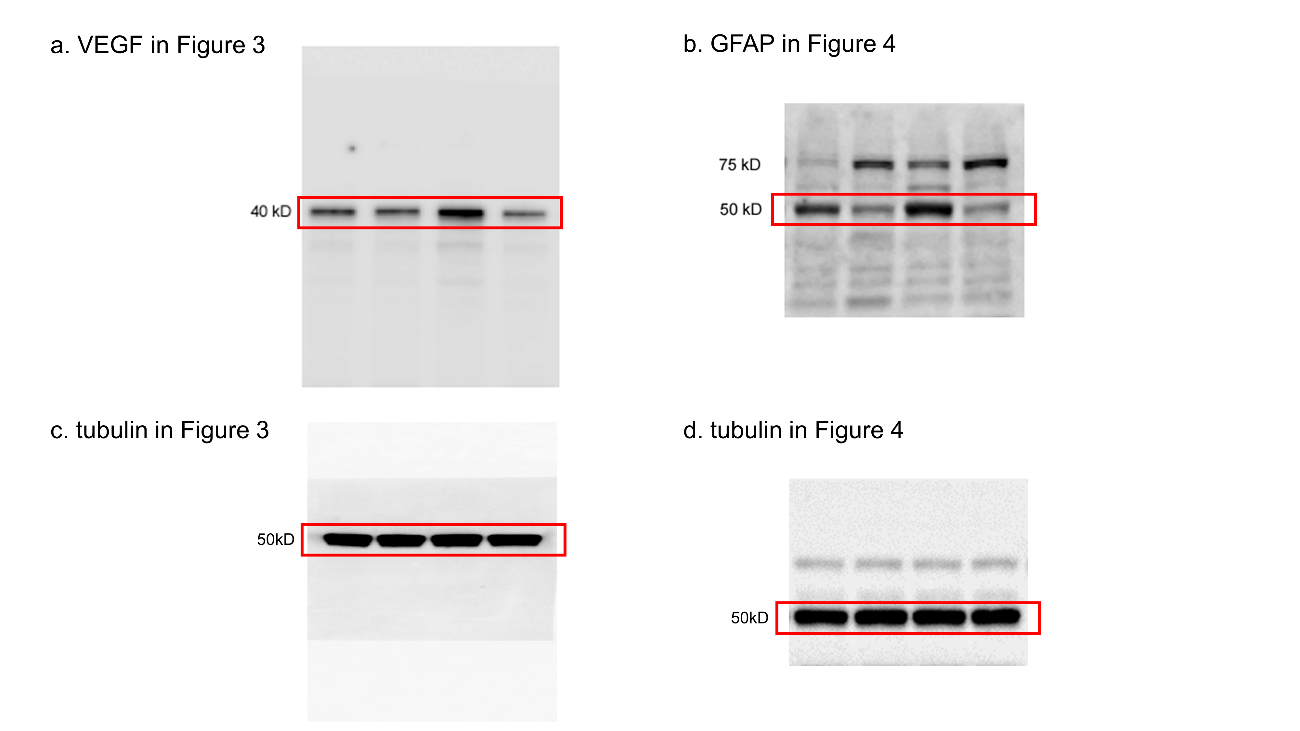

Supplement: Supplementary file 1 — Supplementary Information [file 41598_2021_81482_MOESM1_ESM.docx]
